# *In silico* repurposing of FDA-approved drugs against MEK1: structural and dynamic insights into lung cancer therapeutics

**DOI:** 10.3389/fphar.2025.1619639

**Published:** 2025-08-29

**Authors:** Mohd Shahnawaz Khan, Anas Shamsi, Azna Zuberi, Moyad Shahwan

**Affiliations:** ^1^ Department of Biochemistry, College of Science, King Saud University, Riyadh, Saudi Arabia; ^2^ Centre of Medical and Bio-Allied Health Sciences Research, Ajman University, Ajman, United Arab Emirates; ^3^ Department of Obstetrics and Gynecology, Northwestern University, Chicago, IL, United States; ^4^ Department of Clinical Sciences, College of Pharmacy and Health Sciences, Ajman University, Ajman, United Arab Emirates; ^5^ Center of Excellence in Precision Medicine and Digital Health, Department of Physiology, Faculty of Dentistry, Chulalongkorn University, Bangkok, Thailand

**Keywords:** dual specificity mitogen-activated protein kinase kinase 1, cancer, drug repurposing, small molecule inhibitors, virtual screening

## Abstract

The dual specificity mitogen-activated protein kinase kinase 1 (MEK1) is a critical node in the RAS-RAF-MEK-ERK signaling pathway, frequently dysregulated in cancers due to mutations in upstream regulators. Despite the development of MEK inhibitors, challenges such as on-target toxicities and drug resistance persist that emphasize the need for novel therapeutic strategies. Drug repurposing offers a fast and cost-effective alternative by leveraging existing FDA-approved compounds with established safety profiles. This study employed computational approaches to identify repurposed MEK1 inhibitors through structure-based virtual screening of 3,500 FDA-approved drugs. The MEK1 crystal structure was subjected to molecular docking using InstaDock, followed by biological activity prediction, interaction analysis, and 500-ns molecular dynamics (MD) simulations to assess stability. Radotinib and Alectinib exhibited superior docking scores (−10.5 and −10.2 kcal/mol), outperforming the reference MEK1 inhibitor Selumetinib (−7.2 kcal/mol). MD simulations revealed stable drug complexes, with lower root mean square deviation (RMSD) and fluctuations (RMSF) than Selumetinib. Principal component analysis and free energy landscapes corroborated their conformational stability, suggesting robust binding to MEK1’s allosteric pocket. Radotinib interacted extensively with key residues, including Gly79 and Lys97 at the ATP-binding site, while Alectinib engaged critical residues such as Arg189 and His239. Their superior binding and conformational stability suggest the potential to overcome resistance and toxicity issues associated with existing MEK inhibitors. The structural and dynamic superiority of Radotinib and Alectinib over Selumetinib positions them as promising repurposed MEK1 inhibitors, potentially circumventing the clinical challenges of existing therapies. A limitation of this *in silico* study is the absence of experimental validation, which will be addressed in future work. Experimental validation is essential to confirm their efficacy and safety in MEK1-linked malignancies.

## 1 Introduction

The RAS-RAF-MEK-ERK signalling cascade integrates extracellular signals and coordinates many aspects of cellular physiology, including cell proliferation, survival, differentiation, and apoptosis (Degirmenci et al., 2020). This pathway is dysregulated in more than 30% of human cancers, with upstream components such as RAS, BRAF, or receptor tyrosine kinases being mutated (Bahar et al., 2023). These changes result in constitutive pathway activation that accelerates tumor growth and helps evade apoptosis (Chang et al., 2003). MEK1 (mitogen-activated protein kinase kinase 1) is a dual-specificity kinase that phosphorylates and activates ERK1/2, thus a key component of this cascade (Wang et al., 2022). As such, this enzymatic activity renders MEK1 a pivotal therapeutic target in cancers with mutations that constitute this pathway (Caunt et al., 2015). MEK1 (MAP2K1) and its homolog MEK2 are dual-specificity kinases that phosphorylate ERK1/2 in response to upstream signals, controlling cell proliferation and survival. Aberrant activation of MEK1 (e.g., through BRAF or RAS mutations) is oncogenic, making MEK1 a key cancer target. In the past two decades, an enormous initiative has been made to develop MEK inhibitors (MEKis) (Ram et al., 2023). MEKis often have narrow therapeutic windows and can induce feedback activation of parallel pathways. Type-A allosteric inhibitors that bind to the allosteric site close to the ATP-binding pocket have clinical activities, with a few, e.g., trametinib, selumetinib, approved for some cancers (Roskoski, 2017). Despite their efficacy, therapeutic potential is limited by dose-limiting toxicities, acquired resistance, and narrow therapeutic windows (Ram et al., 2023). These limitations emphasize the need for new inhibitors with better safety and efficacy profiles.

Leveraging efficacy data on existing pharmaceutical agents with known mechanisms of action and side effect spectrums has made drug repurposing an attractive strategy for developing new vaccines and therapeutics, especially as it offers significantly reduced cost and timelines compared to *de novo* drug discovery (Parvathaneni et al., 2019). Repurposing can identify newer usages for already FDA (U.S. Food and Drug Administration)-approved compound classes (Pushpakom et al., 2019). Drug repurposing has successfully identified new uses for kinase inhibitors. Repurposing approaches are being applied to MEK inhibitors outside oncology. For example, trametinib and selumetinib (originally approved for melanoma) have shown efficacy in treating genetic ‘RASopathy’ syndromes, demonstrating the broader potential of MEK-targeted drugs. Similarly, gene expression–based repurposing strategies have uncovered novel drug combinations involving MEK1/2 inhibitors for KRAS-driven lung cancer. In this quest, computational approaches such as structure-based virtual screening and molecular dynamics (MD) simulations have proven powerful tools (Shamsi et al., 2024a). As such, these methods facilitate the extraction of lead drug candidates with tight binding and stability, avoiding the expensive domains of the traditional drug development pathway (Sadybekov and Katritch, 2023). With the successful repurposing of kinase inhibitors for new indications reported elsewhere, this strategy has potential in oncology (Schein, 2021). Even with advances, the structural and dynamic characterization of MEK1-inhibitor complexes is still underexplored. Although the MEK1 allosteric pocket has been clearly defined, the sparse variety of structurally validated binders restrains novel inhibitors from entering the development stage (Di Fruscia et al., 2021). In addition, the native conformation of MEK1 and how it interacts with ligands *in vivo* remains relatively unclear. These gaps highlight the need for detailed biophysical characterization to guide the rational design of potent MEK1 inhibitors.

Here, we implemented a thorough computational pipeline to identify MEK1 inhibitors and screen their potential using FDA-approved drugs. From the virtual screening of 3,500 drugs from DrugBank (Knox et al., 2024) based on the crystal structure of MEK1 (PDB: 7B9L), molecular docking, biological activity prediction, and 500-ns MD simulations were carried out. Root mean square deviation (RMSD), principal component analysis (PCA), and free energy landscapes were further evaluated to assess stability and binding dynamics. Using this approach, we identified Radotinib and Alectinib as high-affinity MEK1 binders, with improved stability over the reference inhibitor Selumetinib. This suggests that repurposed drugs have fewer limitations than current MEKis and provides a rational basis for building practically applicable therapies. Various candidates were identified as being specific to cMYC-transformed cells, and experimental validation may help link computational discovery to therapeutic applications in cancer. This study aims to computationally repurpose FDA-approved drugs as MEK1 inhibitors by integrating structure-based virtual screening, dynamic stability analysis, and thermodynamic profiling to identify candidates with enhanced binding and safety profiles.

## 2 Materials and methods

### 2.1 Molecular docking screening

Virtual screening (VS) is a computational method to identify potential bioactive compounds from large chemical libraries using structure-based molecular docking (Shamsi et al., 2024b). In this study, structure-based VS was performed to evaluate the binding affinities of FDA-approved drugs against MEK1. The crystal structure of MEK1 (PDB ID: 7B9L) was retrieved from the Protein Data Bank (PDB) (Berman et al., 2014). Protein preparation included removal of crystallographic water molecules and co-crystallized ligands, protonation of side chains, addition of Kollman charges, and remodeling of missing residues. Missing residues were modeled using PyMod v3 (Janson and Paiardini, 2021) and processed in AutoDock Tools (Huey et al., 2012). A curated library of 3,500 FDA-approved drugs was sourced from the DrugBank database (Knox et al., 2024) in processed format. Ligand flexibility, including rotatable bonds and bond lengths, was fully permitted during docking. Blind docking was conducted using InstaDock (Mohammad et al., 2021) with a grid encompassing the entire protein structure (dimensions: 85 Å × 78 Å × 67 Å; center coordinates: −21.735, −5.746, 20.809 for the X, Y, and Z-axes, respectively). Docking poses were ranked by binding energy, and top-ranked conformations were selected for further analysis. For each compound, the top-ranked pose with the most favorable binding energy was extracted and analyzed further. Ligands showing the lowest (most negative) docking scores were prioritized for downstream evaluation.

### 2.2 Biological potential and interaction analysis

The biological activities of the selected compounds were predicted using the PASS (Prediction of Activity Spectra for Substances) web server (Filimonov et al., 2014). This tool employs structure-activity relationship (SAR) models trained on diverse biological activity datasets to calculate the probability of a molecule exhibiting specific pharmacological effects, expressed as “probability to be active” (Pa) and “probability to be inactive” (Pi). Compounds with Pa > Pi were prioritized as high-confidence candidates for further investigation. However, PASS predictions do not confirm molecular target specificity and should be interpreted as preliminary, hypothesis-generating tools. The PASS results require subsequent validation through experimental assays or mechanistic modeling. To investigate binding site interactions, molecular docking poses of MEK1–ligand complexes were analyzed using visualization tools. Polar contacts, hydrogen bonds, and hydrophobic interactions were visualized using PyMOL (version 2.5.4) (DeLano, 2002). Discovery Studio Visualizer (v2023) (Visualizer, 2005) was employed to map interactions with residues critical to MEK1’s ATP-binding and catalytic sites for in-depth analysis of binding modes. Key binding residues responsible for ligand stabilization were identified based on distance thresholds and chemical compatibility (e.g., ≤3.5 Å for hydrogen bonds), facilitating insight into potential MEK1 inhibition mechanisms.

### 2.3 MD simulations

Molecular dynamics (MD) simulations were performed to study the dynamic behavior, conformational stability, and flexibility of MEK1 alone and in complex with Alectinib, Radotinib, and Selumetinib. All simulations were carried out with GROMACS 2022.4 (Van Der Spoel et al., 2005) with a CHARMM36m force field (Huang and MacKerell, 2013) using the TIP3P (Mark and Nilsson, 2001) water model. Ligand topology files were created with the CGenFF web server (Zhu, 2019), submitted for geometry optimization, and the charges checked with Avogadro 1.2.0 and our own Python scripts. Four different systems were set up: apo-MEK1, MEK1-Alectinib, MEK1-Radotinib, and MEK1-Selumetinib. All systems were minimized in energy via the steepest descent algorithm (5,000 steps) to remove steric clashes. During this phase, equilibration was conducted in two stages: (1) NVT ensemble (constant number of particles, volume, and temperature) ensemble for 100 ps at 300 K (Berendsen thermostat), and then (2) NPT ensemble (constant number of particles, pressure, and temperature) ensemble for 100 ps at 1 bar (Parrinello-Rahman barostat). Using periodic boundary conditions and a 2-fs time step, production runs were performed for 500 ns. Trajectory analysis performed using GROMACS utilities: the root mean square deviation (RMSD) and the root mean square fluctuation (RMSF) were calculated with *gmx rms* and *gmx rmsf*, respectively, to evaluate the structural stability and residue flexibility. The radius of gyration (*R*g; *gmx gyrate*) and solvent-accessible surface area (SASA; *gmx sasa*) were used to calculate the compactness and solvent accessibility, respectively. We examined the dynamics of the hydrogen bond using *gmx hbond*. All plots were generated using the Grace (XMGRACE) plotting software. These analyses together provided a comprehensive picture of protein-ligand complex behavior, conformational adaptability, and structural stability throughout the simulation timeframe.

### 2.4 Principal component analysis

Principal component analysis (PCA) is a powerful dimensionality reduction and multivariate analysis technique widely used to extract meaningful large-scale motions from MD simulations of biomolecular systems. In the context of this study, PCA was employed to identify and quantify the dominant collective motions and structural fluctuations of MEK1 in its apo and ligand-bound states, thereby elucidating ligand-induced stabilization or flexibility. By filtering out high-frequency random noise, PCA allows the capture of functionally relevant, low-frequency conformational transitions that can be crucial for understanding binding mechanisms and structural stability. The primary reason for employing PCA in this analysis was to explore whether Radotinib and Alectinib modulate MEK1’s conformational dynamics more effectively than Selumetinib. Specifically, PCA helped characterize large-scale domain motions and determine whether these repurposed drugs stabilize MEK1 in a restricted conformational subspace, which may correlate with improved binding affinity and reduced entropic penalties (Papaleo et al., 2009). For PCA calculations, the covariance matrix of atomic positional fluctuations was generated using the Cα atoms from 500-ns MD trajectories, employing the *gmx covar* module in GROMACS. The covariance matrix captures correlated displacements of atom pairs and is defined as:

$$C_{\text{ij}}=<\;\left(x_{i}\;‐\;<\;x_{i}>\right)\;\left(x_{j}\;‐\;<\;x_{j}>\right)\;>$$

where x_i_/x_j_ represents the coordinate of the i^th^/j^th^ atom, and < - > is the ensemble average over the simulation time. Diagonalization of this matrix yields eigenvectors representing directions of motion and corresponding eigenvalues that quantify the variance along those directions. Singular value decomposition (SVD) was applied for matrix diagonalization. This approach enables the identification of ligand-induced effects on protein flexibility by comparing the conformational sampling across systems.

### 2.5 Free energy landscapes

Gibbs free energy landscapes (FELs) were constructed to quantify the thermodynamic stability of MEK1 conformations in apo and ligand-bound states. Projections of the MD trajectories onto the first two principal components (PC1 and PC2), obtained via PCA, were used as reaction coordinates for FEL construction (Papaleo et al., 2009). The conformational probability distribution *P* was computed using the gmx sham module in GROMACS, and the corresponding free energy (Δ*G*) was estimated using the Boltzmann relation:

$$\Delta G=‐k_{B}T\ln\left(P\right)$$

where *P* is the probability density, *k*_*B*_ is the Boltzmann constant, and *T* is the simulation temperature. Energy basins (low Δ*G*) represent thermodynamically stable states, while peaks correspond to high-energy transition states. FELs were visualized as contour plots, with colors scaled from blue (low energy) to red (high energy). This approach elucidated ligand-specific stabilization patterns and identified dominant conformational clusters, revealing how Alectinib and Radotinib modulate MEK1’s energy landscape compared to Selumetinib.

## 3 Results and discussion

### 3.1 Molecular docking screening

Molecular docking serves as a predictive tool to model the interaction between a ligand and its target protein, allowing for identifying molecules with high binding affinity and appropriate conformational orientation within the binding pocket (Muhammed and Aki-Yalcin, 2024). In this study, a virtual screening of 3,500 FDA-approved drugs, curated from the DrugBank repository, was performed against the crystal structure of MEK1 (PDB ID: 7B9L) using the InstaDock platform (Mohammad et al., 2021). The objective was to identify potential MEK1 inhibitors among existing therapeutics that can be repurposed for cancer treatment. Following the docking process, compounds were ranked based on their binding energies, and the top 10 candidates were selected for further analysis (Table 1). Docking scores ranged from −10.1 to −10.8 kcal/mol, indicative of strong binding potential. Notably, all candidates outperformed Selumetinib (−7.2 kcal/mol), underscoring their superior affinity. Importantly, all selected candidates demonstrated significantly better docking scores than the reference MEK1 inhibitor Selumetinib, which exhibited a binding energy of −7.2 kcal/mol. This difference in binding energy suggests that the shortlisted compounds may engage MEK1 more effectively than Selumetinib, potentially translating to improved inhibitory activity. The findings from this docking-based screening highlight the potential of drug repurposing strategies to uncover alternative MEK1 inhibitors that may overcome limitations associated with current therapeutics, such as resistance or toxicity. However, molecular docking represents only the initial filtering step. Further dynamic and energetic assessments, such as MD simulations and binding free energy calculations, are essential to validate these interactions under more physiologically relevant conditions.

**TABLE 1:** Top screened FDA-approved drugs repurposed against MEK1 identified through structure-based virtual screening.

S. No.	Drug	Binding affinity (kcal/mol)	pKi	Ligand efficiency (kcal/mol/non-H atom)	Torsional energy
1	Oxitropium	−10.8	7.92	0.3176	2.8017
2	Delamanid	−10.6	7.77	0.2789	2.8017
3	Fentonium	−10.6	7.77	0.2944	3.113
4	Radotinib	−10.5	7.7	0.2692	2.1791
5	Bictegravir	−10.4	7.63	0.325	1.2452
6	Mosapramine	−10.4	7.63	0.3059	1.2452
7	Alectinib	−10.2	7.48	0.2833	0.9339
8	Fendosal	−10.2	7.48	0.3517	1.5565
9	Pimozide	−10.2	7.48	0.30	2.1791
10	Conivaptan	−10.1	7.41	0.2658	1.2452
11	Selumetinib	−7.2	5.28	0.2667	2.1791

The table lists the top 10 compounds with their respective docking parameters, including binding affinity (kcal/mol), predicted inhibition constant (p*K*i), ligand efficiency (kcal/mol per non-hydrogen atom), and torsional energy values.

### 3.2 Drug profiling and PASS analysis

Drug profiling and predicting other biological activities are key elements of any computational drug repurposing workflow. After the initial molecular docking, the top 10 candidate compounds were subjected to biological activity prediction using the PASS server. The PASS algorithm uses structure‐activity relationship (SAR) models to predict pharmacological effects (Filimonov et al., 2014). The drug profiling for the 10 docked molecules was carried out to evaluate their pharmacological potency in MEK1 inhibition and relevant anticancer properties (Supplementary Table S1). Specifically, Radotinib and Alectinib were identified to have appropriate drug profiles and higher potential for anticancer-related indications. Though other compounds exhibited strong docking potential, their lack of predicted anticancer activity excluded them from further study. The PASS analysis for the selected molecules was carried out to evaluate their pharmacological potency other than MEK1 inhibition (Table 2). Radotinib and Alectinib exhibited greater anticancer potential, with high Pa values across diverse anticancer categories. Importantly, Pa values for both compounds were substantially higher than their respective Pi values, indicative of biological activity. For instance, Radotinib had Pa values particularly high as a growth factor agonist (Pa = 0.797), protein kinase inhibitor (Pa = 0.790), and Bcr-Abl kinase inhibitor (Pa = 0.748), etc. (Zabriskie et al., 2015). Such profiles are consistent with its known mechanism as a tyrosine kinase inhibitor and compatibility with MEK1 targeting.

**TABLE 2:** Predicted pharmacological activities of selected MEK1-binding compounds using PASS (Prediction of Activity Spectra for Substances) analysis.

S. No.	Drug	Pa	Pi	Activity
1	Radotinib	0.797	0.002	Growth factor agonist
		0.790	0.005	Protein kinase inhibitor
		0.748	0.001	Bcr-Abl kinase inhibitor
		0.624	0.009	Angiogenesis inhibitor
		0.430	0.093	Antineoplastic
2	Alectinib	0.276	0.048	Prostate cancer treatment
		0.349	0.126	Antineoplastic
		0.256	0.052	Antineoplastic alkaloid
		0.218	0.060	Antineoplastic (non-small cell lung cancer)
		0.205	0.153	Antimetastatic
3	Selumetinib	0.954	0.000	MAP kinase kinase inhibitor
		0.729	0.021	Antineoplastic
		0.349	0.123	Antiinflammatory
		0.256	0.094	Angiogenesis inhibitor
		0.232	0.163	Autoimmune disorders treatment

The table shows the probability of activity (Pa) and inactivity (Pi) for each compound across relevant biological functions.

Both Radotinib and Alectinib had high Pa values (≫ Pi) for multiple anticancer activities (Table 2). For instance, Radotinib’s top predicted activities include growth factor agonism (Pa≈0.80) and kinase inhibition (Pa≈0.79), consistent with its tyrosine kinase profile. Alectinib likewise showed high Pa for antineoplastic activities. These profiles suggest promising anticancer potential. We further screened Alectinib, an FDA-approved drug for non-small cell lung cancer. We provided favorable probabilities for antineoplastic and antimetastatic activities, demonstrating the program’s utility in onco-logic settings. These results imply that Radotinib and Alectinib show a high binding affinity toward MEK1, coupled with their complementary biological activity profiles consistent with MEK1 inhibition and the treatment of their indicated cancers (Herden and Waller, 2018). Hence, integrating docking data with the biological activity prediction improves the confidence of these hits as potential drug candidates for repurposing. However, PASS predictions are only indicative and based on *in silico* SAR models. Although PASS has a high reported cross-validation accuracy (∼95%) high Pa values do not guarantee an actual biological effect. Thus, over-reliance on PASS is cautioned–experimental assays are needed to confirm the predicted activities.

### 3.3 Interaction analysis

**FIGURE 1:**
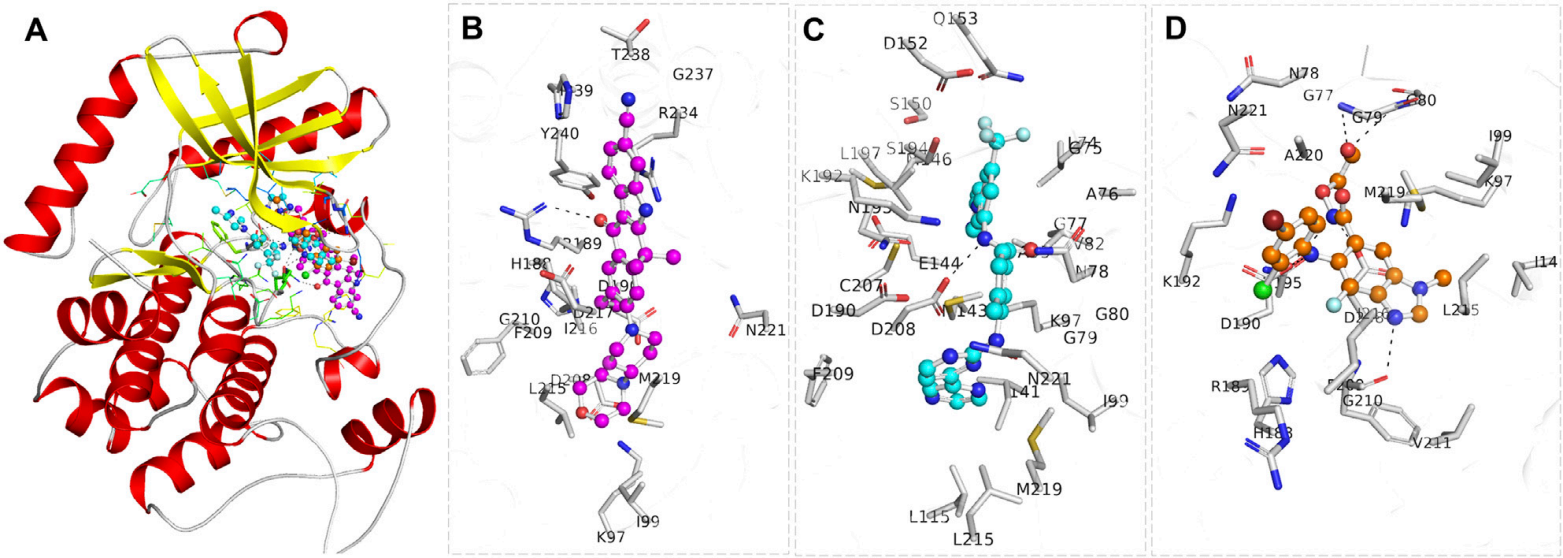
Structural representation of MEK1 in complex with the selected drugs, Alectinib (magenta) and Radotinib (cyan), and the reference MEK1 inhibitor Selumetinib (orange). **(A)** Cartoon representation of the MEK1 structure, with the bound compounds shown within the binding pocket. **(B)** A zoomed-in view of MEK1, illustrating the precise positioning of Alectinib **(C)**, Radotinib, **(D)** Selumetinib. The figure was generated through PyMOL using the structural coordinates from the docking study.

Radotinib and Alectinib were prioritized for further analysis due to their strong docking scores and favorable biological activity profiles. A detailed interaction analysis was conducted for Radotinib, Alectinib, and the reference MEK1 inhibitor Selumetinib using PyMOL and Discovery Studio Visualizer to gain mechanistic insight into their binding modes. Protein–ligand interactions are fundamental to understanding ligand efficacy, as they influence enzymatic activity, signal transduction, protein stability, and drug specificity. Visual representation of the docked complexes revealed that all three compounds, Radotinib (cyan), Alectinib (magenta), and Selumetinib (orange), bound within the MEK1 allosteric pocket located adjacent to the ATP-binding site (Figure 1). Figure 1A shows a cartoon representation of MEK1 highlighting the binding locations of all three ligands. Zoomed-in views in Figures 1B–D illustrate the binding orientations of Alectinib, Radotinib, and Selumetinib, respectively. The ligands predominantly interacted with key regions involved in ATP and inhibitor binding, including the Leu74–Val82 cleft, Lys97 (ATP/inhibitor-binding site), Asp190 (proton acceptor active site), and the Asp208–Val211 segment, indicating their potential to modulate MEK1 activity through direct interaction with catalytically relevant residues.

**FIGURE 2:**
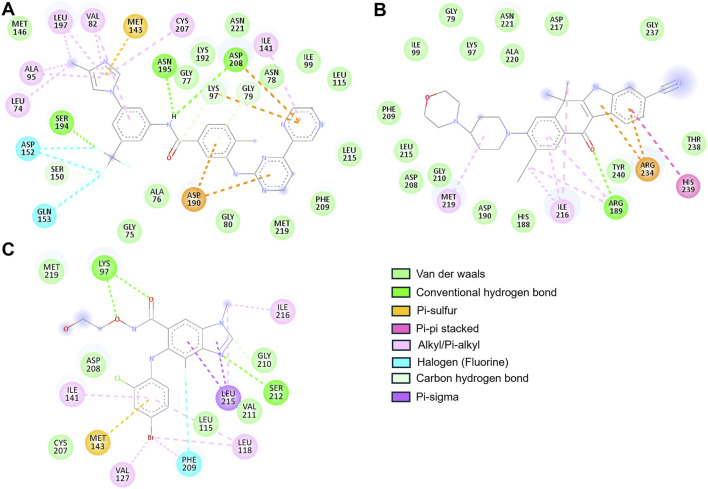
2D interaction maps illustrating binding residues of MEK1 and their interactions with **(A)** Radotinib, **(B)** Alectinib, and **(C)** Selumetinib.

The two-dimensional interaction maps provided a detailed account of the molecular interactions (Figure 2). Alectinib displayed a stable and meaningful interaction pattern. It formed a hydrogen bond with Arg189 and engaged in pi-pi stacking with His239. A pi–sulfur interaction was observed with Arg234, while hydrophobic alkyl and pi–alkyl interactions involved Arg189, Ile216, and Met219. Van der Waals interactions further anchored the ligand via contacts with residues such as Gly79, Lys97, Ile99, His188, Asp190, Asp208, Phe209, Gly210, Leu215, Asp217, Ala220, Asn221, Gly237, Thr238, and Tyr240 (Figure 2A). Notably, Alectinib interacted with Gly79, Lys97, and Asp190, which are part of the catalytic core, further suggesting its therapeutic relevance in MEK1 inhibition (Di Fruscia et al., 2021). At the same time, Radotinib also demonstrated a rich interaction profile, forming hydrogen bonds with residues Gly79, Lys97, Ser150, Ser194, Asn195, and Asp208. Additionally, it exhibited halogen interactions (e.g., fluorine) with Asp152, Gln153, and Ser194, along with pi–sulfur interactions involving Lys97, Asp190, Asp208, and Met143. Radotinib’s halogen bond with Asp152 may enhance binding specificity by mimicking ATP’s phosphate interactions. Hydrophobic contacts such as alkyl and pi–alkyl interactions were observed with Leu74, Val82, Ala95, Ile141, Leu197, and Cys207, while van der Waals forces stabilized the complex through interactions with Gly75, Ala76, Gly77, Asn78, Gly80, Ile99, Leu115, Met146, Lys192, Phe209, Leu215, Met219, and Asn221 (Figure 2B).

These extensive interactions, especially with residues in the ATP-binding cleft and active site, underscore Radotinib’s potential as a robust MEK1 inhibitor. In contrast, Selumetinib, the reference compound, is a non-ATP-competitive MEK inhibitor, showed comparatively limited interactions. It formed hydrogen bonds with Lys97 and Ser212 and engaged in pi–sigma and pi–sulfur interactions with Leu215 and Met143, respectively. Hydrophobic contacts included interactions with Leu118, Val127, Ile141, Ile216, and Leu215, while van der Waals interactions occurred with Leu115, Cys207, Asp208, Gly210, Val211, and Met219 (Figure 2C). Among the residues in the ATP-binding region, Selumetinib directly interacted with Lys97, indicating a more constrained binding profile compared to Radotinib and Alectinib. Both Radotinib and Alectinib demonstrated broader and more diverse interaction networks within the MEK1 binding pocket than the reference inhibitor. Their ability to engage multiple key residues, particularly at the ATP-binding and allosteric sites, reinforces their potential as strong MEK1 inhibitors. These results provided a structural rationale for their selection and prompted further assessment using MD simulations to evaluate binding stability and dynamic behavior under physiological conditions.

### 3.4 MD simulation analysis

MD simulations have become an indispensable tool in molecular biology and drug discovery, enabling high-resolution insights into the atomic-level behavior of proteins and their interactions with ligands (Singh and Singh, 2020). In this study, MD simulations were employed to investigate the dynamic behavior of MEK1 in its apo form and in complex with three compounds: Radotinib, Alectinib, and Selumetinib. The simulations were conducted using GROMACS 2022.4 on a Linux platform, with a total simulation time of 500 nanoseconds for each system. Trajectory analysis of these simulations provided multiple parameters to assess the dynamic behavior and stability of MEK1 and its complexes. Three energy components were initially evaluated: potential, kinetic, and total energy. The potential energies (in kJ/mol) for the MEK1, MEK1-Alectinib, MEK1-Radotinib, and MEK1-Selumetinib systems were found to be −1,058,300, −742,093, −743,103, and −742,440, respectively. The corresponding kinetic energy values were 202,973, 145,759, 145,871, and 145,765 kJ/mol, while the total energy values were −855,325, −596,335, −597,231, and −596,675 kJ/mol, respectively. These energy profiles suggest that all three complexes exhibit stable energy states, with MEK1 retaining its structural integrity even upon drug binding. Notably, the drug-bound systems showed consistently lower total and potential energy values than the apo form, indicating enhanced thermodynamic stability upon ligand binding.

#### 3.4.1 Stability prediction by RMSD and RMSF calculations

**FIGURE 3:**
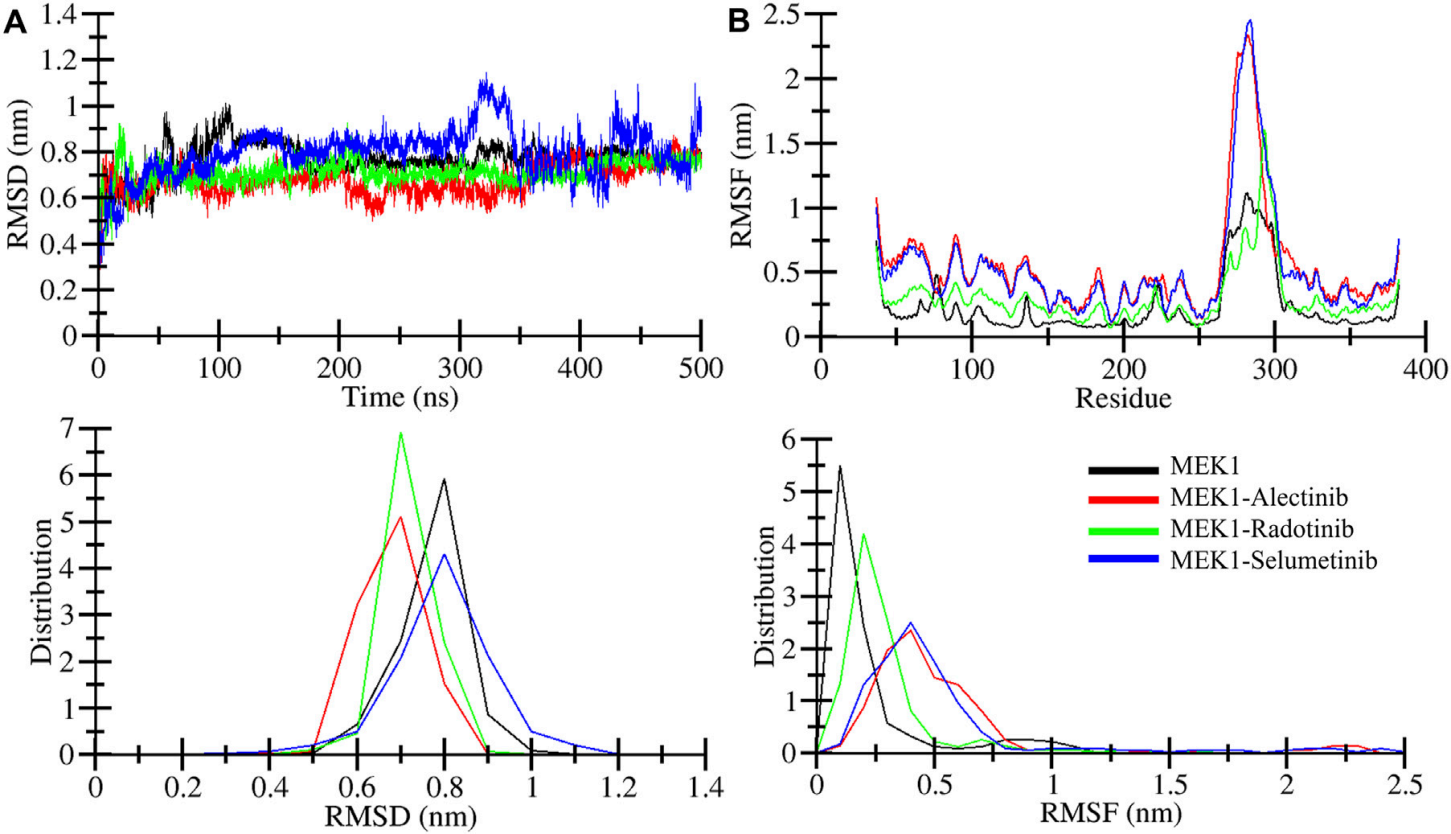
Structural dynamics analysis. **(A)** Structural deviation quantified for MEK1, MEK1-Alectinib, MEK1-Radotinib, and MEK1-Selumetinib complexes from 500ns simulation trajectories. **(B)** Individual residual fluctuation during a 500 ns simulation calculated for MEK1, MEK1-Alectinib, MEK1-Radotinib, and MEK1-Selumetinib complexes. The lower panel figures depict the distribution of RMSD and RMSF.

Root mean square deviation (RMSD) is utilized to estimate the difference between the backbones of a protein from its starting conformation to its final conformation (Maiorov and Crippen, 1994). The stability of the protein structure associated with its native conformation can be calculated by the deviations observed during its simulation. The fewer deviations, the more stable the protein structure, or *vice versa*. The Radotinib- and Alectinib-bound complexes showed lower average RMSD and RMSF than the reference Selumetinib complex (see Table 3). Radotinib complex RMSD plateaued around 0.71 nm and Alectinib around 0.67 nm, both below Selumetinib’s ∼0.79 nm (Table 3). Similarly, Radotinib yielded the lowest average RMSF (∼0.30 nm vs. 0.52 nm for Selumetinib), indicating reduced flexibility. These results (Table 3) imply enhanced stability for the repurposed drugs. The RMSD values of MEK1-Alectinib and MEK1-Radotinib complexes were lower than those of MEK1 protein and reference MEK1-Selumetinib complexes. The generated RMSD plot against time in nanoseconds of all complexes is shown in Figure 3A. The Selumetinib-bound MEK1 (blue trace) showed larger RMSD fluctuations than the other complexes than the other plots throughout the simulation. MEK1-Alectinib and MEK1-Radotinib complex plots were in equilibrium state after initial adjustment till the end of the simulation. The PDF plot also shows different distribution points of each system.

**TABLE 3:** Average MD parameters for MEK1 and ligand-bound complexes.

Complexes	RMSD (nm)	RMSF (nm)	*R*g (nm)	SASA (nm)	Intra H-bonds
MEK1	0.76	0.23	2.14	189.3	228
MEK1-Alectinib	0.67	0.54	2.24	200.1	225
MEK1-Radotinib	0.71	0.30	2.14	185.2	240
MEK1-Selumetinib	0.79	0.52	2.19	195.8	234

RMSD, root-mean-square deviation; RMSF, root-mean-square fluctuation; Rg, radius of gyration; SASA, solvent-accessible surface area; Intra H-bonds, number of intramolecular hydrogen bonds.

Root mean square fluctuation (RMSF) measures the protein particle (residues) fluctuations over time (Shamsi et al., 2024b). Here, we analyzed RMSF for MEK1, MEK1-Alectinib, MEK1-Radotinib, and MEK1-Selumetinib complexes over 500 nanoseconds; the generated RMSF plot is represented in Figure 3B. The plot indicates random fluctuations of the MEK1-Alectinib complex, which overlaps with the reference MEK1-Selumetinib complex, while the MEK1-Radotinib complex shows lower fluctuations. The higher fluctuation was observed between 270 and 300 residues for MEK1-Alectinib and MEK1-Selumetinib complex. The average RMSF value for MEK1, MEK1-Alectinib, MEK1-Radotinib, and MEK1-Selumetinib complexes was 0.23 nm, 0.54 nm, 0.30 nm, and 0.52 nm, respectively (Table 3). Maximum RMSF reach for MEK1, MEK1-Alectinib, MEK1-Radotinib, and MEK1-Selumetinib complexes were 1.11 nm, 2.33 nm, 1.60 nm, and 2.45 nm, respectively. Compared to the reference MEK1-Selumetinib complex, MEK1-Alectinib and MEK1-Radotinib complexes were found to exhibit lower residual fluctuations. The PDF plots also show varying points of the RMSF values with a similar trend. These reduced RMSD and RMSF values reflect enhanced conformational stability, potentially contributing to improved inhibitory action.

#### 3.4.2 Compactness and folding mechanism assessment by Rg and SASA

**FIGURE 4:**
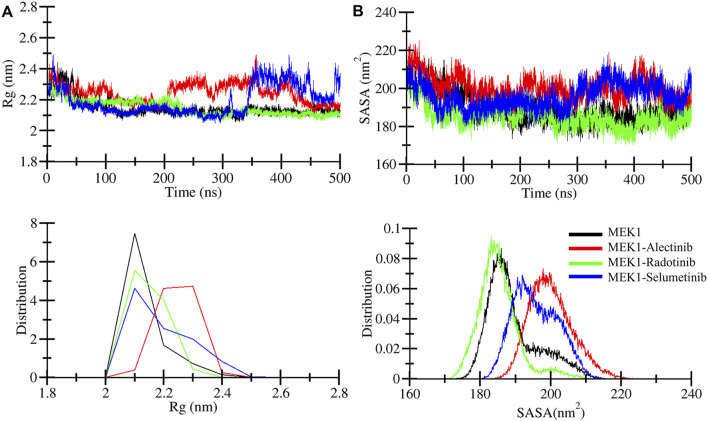
Structural compactness analysis. **(A)** Structural compactness quantified for MEK1, MEK1-Alectinib, MEK1-Radotinib, and MEK1-Selumetinib complexes from 500 ns simulation trajectories. **(B)** Surface area assessment during a 500 ns simulation was calculated for MEK1, MEK1-Alectinib, MEK1-Radotinib, and MEK1-Selumetinib complexes. The lower panel figures depict the distribution of *R*g and SASA.

Radius of gyration (*R*g) analysis was performed to illustrate the compactness of the MEK1 protein and to estimate the overall size of the MEK1 protein. *R*g provides a detailed description about mass distribution around the molecule’s center of mass and its dynamic structural properties (Lobanov et al., 2008). We can access structural expansion and contraction by calculating *R*g during simulation time. Here we performed *R*g analysis of MEK1, MEK1-Alectinib, MEK1-Radotinib, and MEK1-Selumetinib complexes over 500 ns time and generated the *R*g plot depicted in Figure 4A. A marginal fluctuation in the MEK1-Alectinib complex plot (red) was observed after 200 ns. However, higher fluctuation was observed in the reference inhibitor MEK1-Selumetinib complex plot (blue) from 340 ns onwards, which was higher than the MEK1-Alectinib complex. The MEK1-Alectinib complex plot was in an equilibrium state throughout the simulation. The average *R*g of MEK1, MEK1-Alectinib, MEK1-Radotinib, and MEK1-Selumetinib complexes were 2.14 nm, 2.24 nm, 2.14 nm, and 2.19 nm, respectively (Table 3). Maximum *R*g values of MEK1, MEK1-Alectinib, MEK1-Radotinib, and MEK1-Selumetinib complexes were 2.44 nm, 2.49 nm, 2.37 nm, and 2.49 nm, respectively. The generated plots, calculated values, and distribution plot as a PDF revealed that the MEK1 structure retained its compactness after binding the drugs.

Solvent accessible surface area (SASA) refers to the part of the protein surface that is available to contact by solvent molecules during MD simulation. SASA is an important parameter to elucidate folding patterns, stability, and interactions with other molecules, such as water, ions, etc. We computed SASA for MEK1, MEK1-Alectinib, MEK1-Radotinib, and MEK1-Selumetinib complexes and generated a plot in Figure 4B. In the SASA plot, the MEK1-Alectinib complex shows higher fluctuations in comparison to other complexes. The MEK1-Alectinib complex shows SASA’s downward trend and equilibrium throughout the simulation. Average SASA values for MEK1, MEK1-Alectinib, MEK1-Radotinib, and MEK1-Selumetinib complexes were 189.3 nm^2^, 200.1 nm^2^, 185.2 nm^2^, and 195.8 nm^2^ respectively (Table 3). The findings indicate that the MEK1-Alectinib and reference MEK1-Selumetinib complexes revealed a wider surface area occupied by solvents during the simulation. The PDF plot also indicates the SASA distribution point of each system, in which the MEK1-Alectinib complex showed a wider area, but it did not have a worse impact on folding and stability.

#### 3.4.3 Stability prediction by hydrogen bonds assessment

**FIGURE 5:**
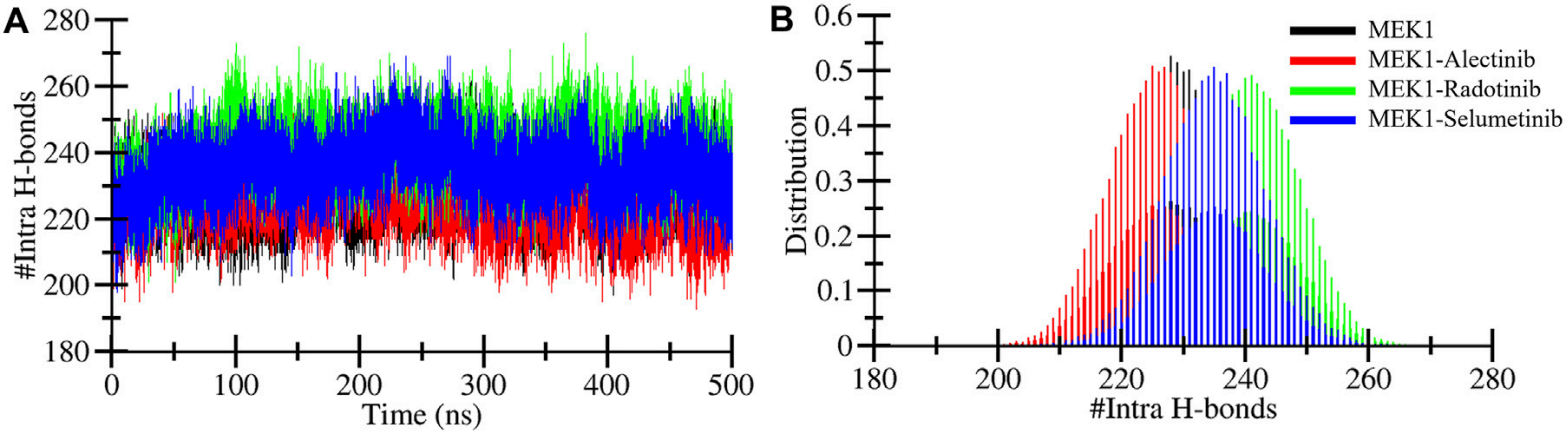
Intramolecular hydrogen bonds in MEK1. **(A)** Intramolecular hydrogen bonds determination for MEK1, MEK1-Alectinib, MEK1-Radotinib, and MEK1-Selumetinib complexes. **(B)** Intermolecular hydrogen bond computed between the MEK1-Alectinib complex. The lower panel figures depict the distribution of Intramolecular and intermolecular hydrogen bonds.

Hydrogen bonds are considered for their significance in protein stability measurement during MD simulations (Hubbard and Haider, 2010). The hydrogen bonds within proteins provide stable conformations and shape and influence biological function. The computed intramolecular hydrogen bonds plot of MEK1, MEK1-Alectinib, MEK1-Radotinib, and MEK1-Selumetinib complexes is shown in Figure 5. The plot demonstrates the making and breaking of an intramolecular hydrogen bond pattern over 500 ns. As the plot indicates, the MEK1-Radotinib complex shows more bond formations between 100 and 400 ns of simulation (Figure 5A). The average intramolecular hydrogen bonds for MEK1, MEK1-Alectinib, MEK1-Radotinib, and MEK1-Selumetinib complexes were 228, 225, 240, and 234, respectively. The MEK1-Alectinib complex broke three hydrogen bonds, while the MEK1-Radotinib complex formed 12 new intramolecular hydrogen bonds. Maximum intra-molecular hydrogen bonds for MEK1, MEK1-Alectinib, MEK1-Radotinib, and MEK1-Selumetinib complexes were 262, 257, 276, and 269, respectively. The PDF plot and calculated number of bonds show stronger stability of the MEK1-Radotinib complex (Figure 5B).

**FIGURE 6:**
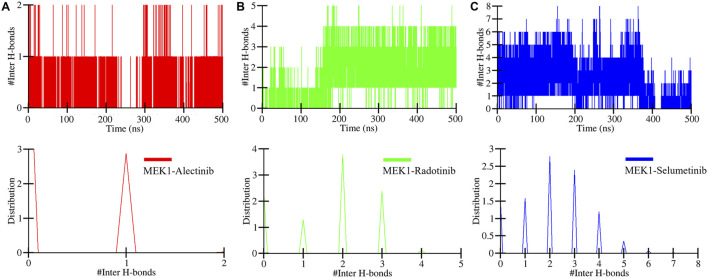
Intermolecular hydrogen bonds plots of **(A)** MEK1-Alectinib, **(B)** MEK1-Radotinib, and **(C)** MEK1-Selumetinib complex. The lower panels show the distribution of intermolecular hydrogen bonds.

The intermolecular hydrogen bonds were also determined during the 500 ns MD simulation. This significantly plays a crucial role in the stability of protein-ligand complexes, their function, and binding energy (Bitencourt-Ferreira et al., 2019). The computed intermolecular hydrogen plot of MEK1-Alectinib, MEK1-Radotinib, and MEK1-Selumetinib complexes is displayed in Figure 6. The MEK1-Alectinib, MEK1-Radotinib, and MEK1-Selumetinib complexes formed 1–2, 1–5, and 1–8 intermolecular hydrogen bonds, respectively (Figures 6A–C). The lower panel plot shows the distribution of intermolecular hydrogen bonds between the complex during simulation. Notably, Selumetinib’s higher H-bond count did not translate to superior stability, as reflected in its elevated RMSD/RMSF values (Table 3). This suggests that H-bond quality (e.g., bond length/angle consistency, partner residues) may outweigh quantity in stabilizing MEK1-inhibitor complexes. For instance, Radotinib’s stable H-bonds with catalytic residues likely restrict ATP-pocket dynamics, while Selumetinib’s transient interactions with peripheral residues (e.g., Ser212) permit conformational flexibility. Overall, the hydrogen bonds assessment suggested that the MEK1 remained stable during the simulation when interacting with the drugs.

#### 3.4.4 MEK1 secondary structure elements profile

**FIGURE 7:**
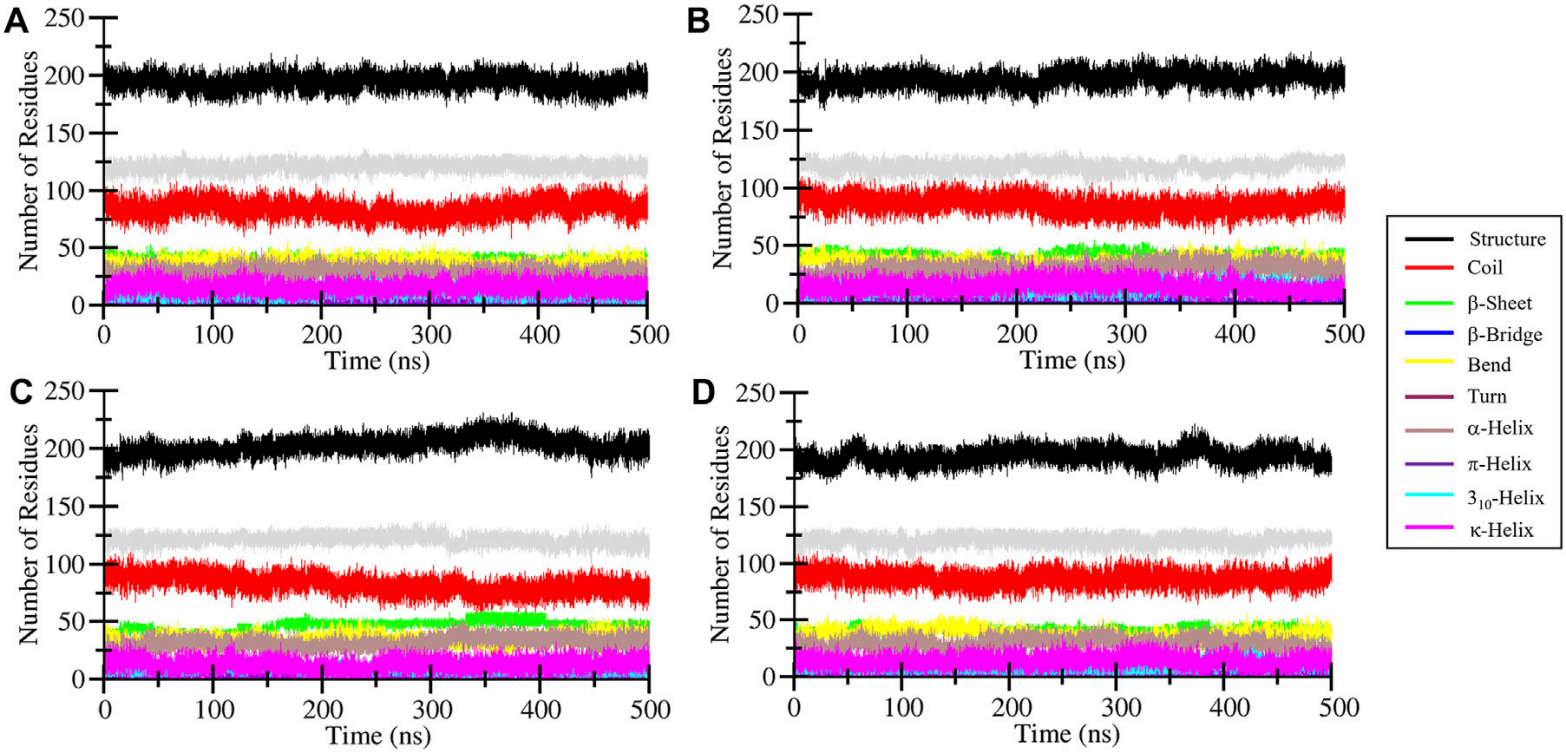
Time-resolved analysis of secondary structure elements in MEK1 and its ligand-bound complexes during 500-ns MD simulations. Secondary structure assignments were determined using the DSSP algorithm and are represented for **(A)** apo MEK1, **(B)** MEK1-Alectinib, **(C)** MEK1-Radotinib, and **(D)** MEK1-Selumetinib complexes. Color-coded bands depict the dynamic distribution of helices, sheets, turns, and coils over time.

The secondary structure content of MEK1 protein before and after Alectinib, Radotinib, and Selumetinib binding was analyzed over time. The GROMACS-based Dictionary of Secondary Structure of Proteins (DSSP) tool (Gorelov et al., 2024) was utilized to break secondary structure assignments (helix, sheet, turn, etc.) at the residue level for each time step. It allowed us to visualize and quantify the secondary structure content in a meaningful form. The generated secondary structure assignment plot is depicted in Figure 7 and shown by different color shades. A few random minor fluctuations were observed over time in MEK1, MEK1-Alectinib, MEK1-Radotinib, and MEK1-Selumetinib plots (Figures 7A–D). The quantitative values given in Table 4 show increasing residual involvement in the structure of MEK1-Radotinib complex. The minimal decrement in bend formation of MEK1-Alectinib, MEK1-Radotinib complex was seen, and the β-bridge was consistent. Overall, no significant residual reduction was observed in any elements of the MEK1 secondary structure after drug interaction throughout the 500 ns simulation. The findings recommended that MEK1 was in a stable conformation state.

**TABLE 4:** Secondary structure composition (fraction of residues) in MEK1 and complexes after 500 ns MD (calculated by DSSP).

Complexes	Structure	Coil	β-sheet	β-bridge	Bend	Turn	α-helix	Pi-helix	3_10_-helix	PPII-helix
MEK1	0.56	0.24	0.12	0.01	0.11	0.08	0.35	0.01	0.03	0.05
MEK1-Alectinib	0.56	0.25	0.12	0.01	0.10	0.09	0.34	0.00	0.04	0.04
MEK1-Radotinib	0.59	0.24	0.14	0.01	0.10	0.09	0.35	0.01	0.03	0.04
MEK1-Selumetinib	0.56	0.25	0.12	0.01	0.11	0.08	0.35	0.01	0.03	0.04

### 3.5 Principal component analysis

**FIGURE 8:**
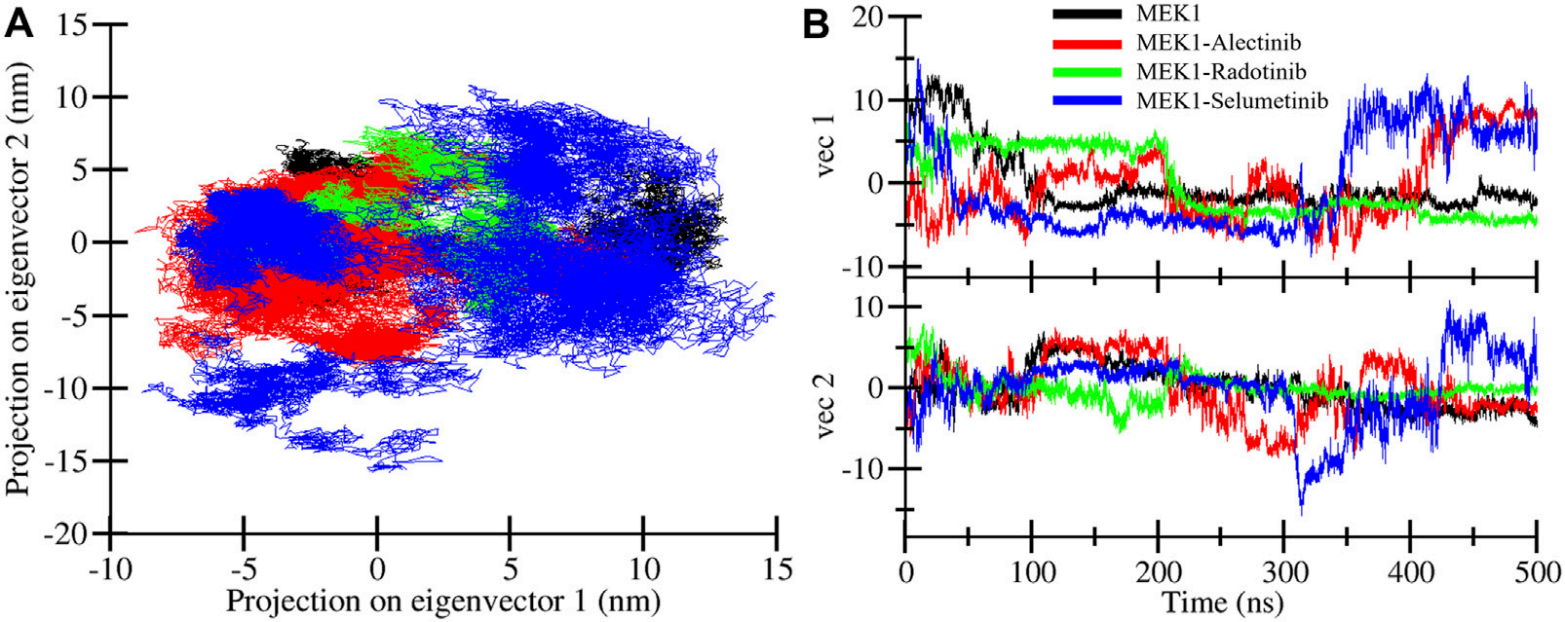
Principal component plots. **(A)** Overlapped PCA plot of MEK1, MEK1-Alectinib, MEK1-Radotinib, and MEK1-Selumetinib complexes. **(B)** Time-dependent eigenvector assessment for MEK1, MEK1-Alectinib, MEK1-Radotinib, and MEK1-Selumetinib complexes.

To illustrate the most dominant motion of the MEK1 protein after binding of Alectinib, Radotinib, and Selumetinib drugs during MD simulation, PCA was utilized on the coordinate covariance matrix extracted from trajectories of a 500 ns MD simulation. Most of the structural motions are captured through the first two principal components; thus, we performed PCA analysis of MEK1, MEK1-Alectinib, MEK1-Radotinib, and MEK1-Selumetinib complexes. Figure 8 displays the superimposed PCA plot for MEK1, MEK1-Alectinib, MEK1-Radotinib, and MEK1-Selumetinib complexes of the first two principal components. As the PCA plot shows, each complex occupied a different range of vibrational space, which indicates different motion patterns. The MEK1-Alectinib and MEK1-Radotinib complexes covered less vibrational space, while the reference MEK1-Selumetinib complex was dispersed and occupied a larger area. The area of motion covered by MEK1 at PC1 −4 nm to 12.9 nm at PC2 −5.2 nm to 7.0 nm, MEK1-Alectinib complex at PC1 −9.1 nm to 10.3 nm at PC2 −8.5 nm to 7.4 nm, MEK1-Radotinib complex at PC1 −5.3 to 7.2 at PC2 5.5 nm–8.0 nm and MEK1-Selumetinib complex at PC1 −8.7 nm to 14.7 nm at PC2 15.5 nm–10.9 nm (Figure 8A).

The results showed that the first PC1 carried most of the motion. For example, PC1 had an eigenvalue of ∼15.2 (accounting for roughly 71% of the total variance), and the second component (PC2) had an eigenvalue of ∼3.4 (∼16% of variance). Together, PC1 and PC2 explained 87% of the total conformational fluctuation (dominant motions). These values indicate that nearly all collective motion is captured by the first two modes, which is consistent with typical MD PCA results where PC1 dominates. The calculated values and superimposed PCA plot show that MEK1-Alectinib and MEK1-Radotinib complex display a cluster and compact type of motion compared to MEK1-Selumetinib complex. The time‐dependent eigenvector traces similarly show that projections onto PC1 and PC2 remain relatively stable for the Alectinib and Radotinib cases, whereas the Selumetinib complex exhibits larger fluctuations (Figure 8B). Together, the high percentage of variance in PC1+PC2 and the compact clusters for Alectinib/Radotinib indicate that most conformational variability is confined to a few dominant motions.

### 3.6 Free energy landscape analysis

**FIGURE 9:**
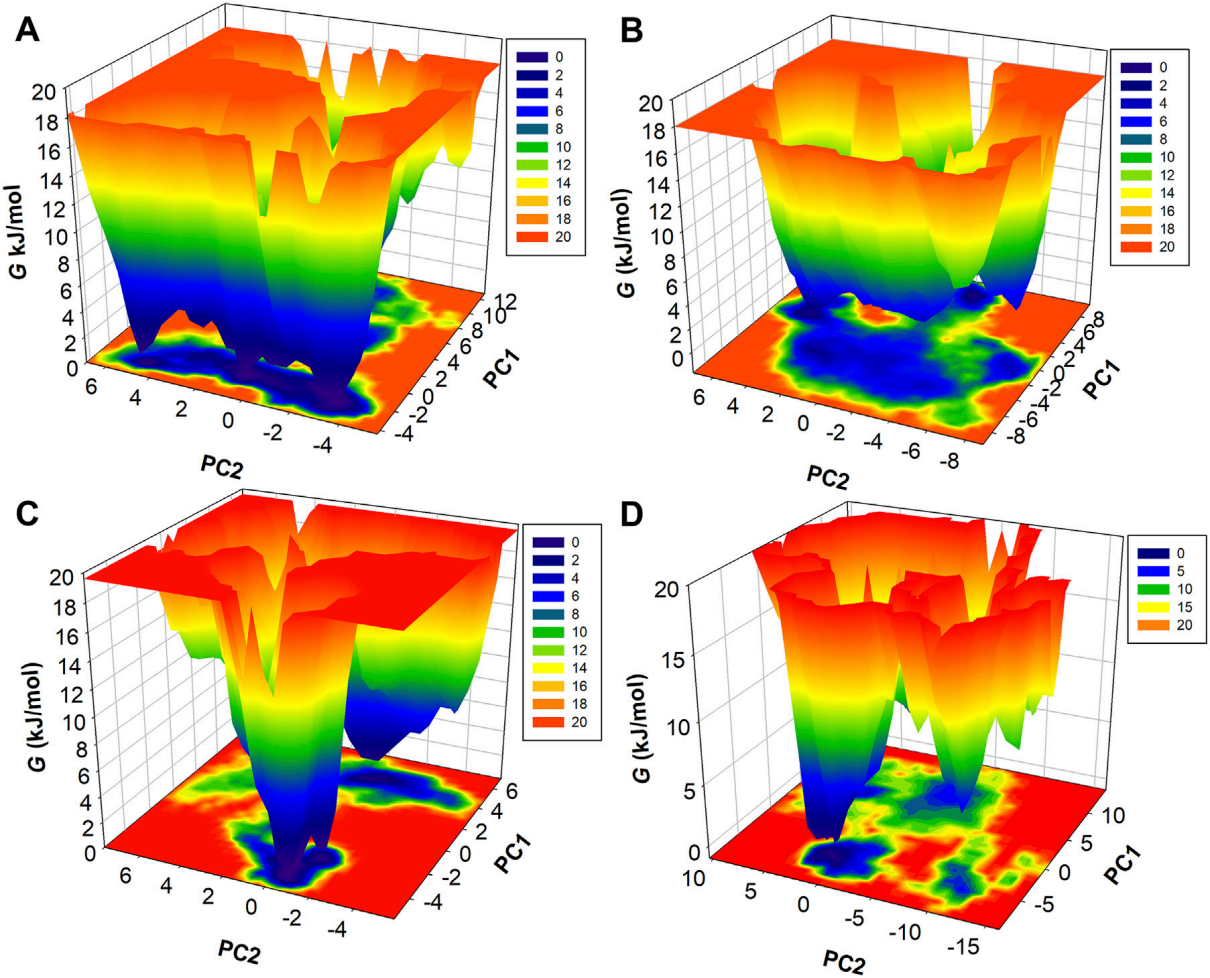
Three-dimensional FEL maps of **(A)** MEK1, **(B)** MEK1-Alectinib, **(C)** MEK1-Radotinib, and **(D)** MEK1-Selumetinib complex.

Further to describe the structural dynamics of MEK1, MEK1-Alectinib, MEK1-Radotinib, and MEK1-Selumetinib complexes, the metastable conformations state, which were dominant during the simulation, were fetched from the FEL. Different energy states are indicated by different colors, from highest to lowest, denoted by red to blue. The FEL of MEK1, MEK1-Alectinib, MEK1-Radotinib, and MEK1-Selumetinib complexes are given in Figure 9. The MEK1 FEL map had one long blue energy minimum and multiple energy funnels linked (Figure 9A). The MEK1-Alectinib complex map shows a broader area of blue basin with combined energy funnels (Figure 9B). In contrast, the MEK1-Radotinib complex had two blue energy minima and two separate energy funnels, which indicate two different folding states (Figure 9C). The reference MEK1-Selumetinib complex map shows multiple energy funnels, which are separated from each other, demonstrating different folding states (Figure 9D). The resulting PCA analysis and FEL calculation suggested that MEK1-Alectinib and MEK1-Radotinib complexes were in stable form during simulations. These observations imply that Radotinib and Alectinib modulate the conformational flexibility of MEK1 more effectively than Selumetinib. Their distinct energy basins and stable low-energy states suggest a reduced likelihood of unfavorable conformational transitions. This thermodynamic stability further supports their potential as robust MEK1 inhibitors. Collectively, the FEL and PCA analyses reinforce the structural reliability and inhibitory promise of these repurposed compounds.

Importantly, both Radotinib and Alectinib are approved kinase inhibitors with known off-target profiles. Radotinib is a BCR-ABL1 tyrosine kinase inhibitor that also inhibits DDR, EPHB, LYN, and PDGFR kinases at low-nanomolar levels (Zabriskie et al., 2015; Liu et al., 2022). Alectinib is an ALK inhibitor with potent activity against RET kinase (IC50 ≈ 1.9 nM for ALK, 4.8 nM for RET (Kodama et al., 2014). These multi-kinase activities mean that repurposing them for MEK1 could bring unintended effects via their original targets. Therefore, future work should include broad kinase profiling to confirm MEK1 selectivity and assess off-target risks. A key limitation of this study is its purely *in silico* nature. All findings must be validated experimentally; in future work, we plan *in vitro* kinase assays, cell-based MEK1 activity tests, and *in vivo* studies to confirm efficacy. Additionally, biochemical assays will be used to determine the selectivity profiles of Radotinib and Alectinib against a panel of kinases.

## 4 Conclusion

The RAS-RAF-MEK-ERK signaling cascade is a critical pathway implicated in various cancers, with MEK1 serving as a key therapeutic target due to its role in activating ERK1/2. Although several MEK1 inhibitors have been approved, their clinical utility is limited by drug resistance, toxicity, and narrow therapeutic windows, necessitating the search for alternative inhibitors with improved profiles. This study employed a comprehensive computational drug repurposing pipeline to screen 3,500 FDA-approved compounds against MEK1. Radotinib and Alectinib emerged as promising candidates, exhibiting significantly better binding affinity and interaction profiles than the reference inhibitor Selumetinib. Detailed molecular dynamics simulations demonstrated that both compounds formed stable complexes with MEK1, maintaining structural integrity and favorable biophysical properties throughout 500-ns trajectories. Principal component and free energy landscape analyses further confirmed their ability to stabilize MEK1 in thermodynamically favorable conformations. Altogether, these findings highlight the potential of Radotinib and Alectinib as effective MEK1 inhibitors, warranting further *in vitro* and *in vivo* validation. In future work, one can perform biochemical MEK1 inhibition assays and cancer cell studies with Radotinib and Alectinib to verify their efficacy. Selectivity profiling across a panel of kinases should also be conducted to ensure their action is specific to MEK1. Overall, our study highlights the potential of structure-based drug repurposing to streamline cancer therapeutic development and paves the way for preclinical validation of Radotinib and Alectinib as MEK1-targeted agents. We stress that all predictions here are preliminary; cell-based and animal experiments are needed to confirm these repurposing leads.

## Data Availability

The original contributions presented in the study are included in the article/Supplementary Material, further inquiries can be directed to the corresponding authors.

## Abbreviations

MD, molecular dynamics; PCA, principal component analysis; FEL, free energy landscape; RMSD, root mean square deviation; RMSF, root mean square fluctuation; Rg, radius of gyration; SASA, solvent-accessible surface area; ATP, adenosine triphosphate; PASS, prediction of activity spectra for substances; Pa, probability to be active; Pi, probability to be inactive; PC, principal component; Δ*G*, Gibbs free energy; kB, Boltzmann constant; T, temperature in kelvin; SVD, singular value decomposition; PDB, Protein Data Bank; VS, virtual screening; SAR, structure‐activity relationship; NVT, constant number of particles, volume, and temperature; NPT, constant number of particles, pressure, and temperature; PME, particle mesh Ewald; LINCS, linear constraint solver; TKI, tyrosine kinase inhibitor; MEK1, mitogen-activated protein kinase kinase 1; FDA, U.S. Food and Drug Administration; Cα, alpha carbon atom; CGenFF, CHARMM general force field; CHARMM36m, Chemistry at HARvard Macromolecular Mechanics 36m force field; PPII, polyproline II helix; 310-helix, three residues per turn helix.
