# Supplementary material for: In silico repurposing of FDA-approved drugs against MEK1: structural and dynamic insights into lung cancer therapeutics
Source: Front Pharmacol. 2025 Aug 29;16:1619639. doi: 10.3389/fphar.2025.1619639 (PMC12426037; doi:10.3389/fphar.2025.1619639)
Supplement: Supplementary file 1 [file Table1.docx]

**Running Title:** *Repurposing Radotinib and Alectinib as MEK1 Inhibitors*

**In Silico Repurposing of FDA-Approved Drugs Against MEK1: Structural and Dynamic Insights into lung Cancer Therapeutics**

Mohd Shahnawaz Khan^1#^, Anas Shamsi^2*^, Azna Zuberi^3^, Moyad Shahwan^4,5^

*^1^Department of Biochemistry, College of Science, King Saud University, Riyadh, Kingdom of Saudi Arabia. Email:* [*moskhan@ksu.edu.sa*](mailto:moskhan@ksu.edu.sa)

*^2^Center for Medical and Bio-Allied Health Sciences Research, Ajman University, UAE. Email:* [*anas.shamsi18@gmail.com*](mailto:anas.shamsi18@gmail.com)

*^3^Northwestern University, Chicago, United States of America. Email:* [*azna.zuberi@northwestern.edu*](mailto:azna.zuberi@northwestern.edu)

*^4^Department of Clinical Sciences, College of Pharmacy and Health Sciences, Ajman University, Ajman, UAE. Email:* [*moyad76@hotmail.com*](mailto:moyad76@hotmail.com)

*^5^Center of Excellence in Precision Medicine and Digital Health, Department of Physiology, Faculty of Dentistry, Chulalongkorn University, Bangkok, Thailand.*

***Corresponding Author**

**Anas Shamsi**, PhD

Centre of Medical and Bio-allied Health Sciences Research

Ajman University

United Arab Emirates.

Email: [anas.shamsi18@gmail.com](mailto:anas.shamsi18@gmail.com)

**#Co Corresponding Author**

Mohd Shahnawaz Khan, PhD

King Saud University

Saudi Arabia

Email: [moskhan@ksu.edu.sa](mailto:moskhan@ksu.edu.sa)

**Supplementary Table S1:** Molecular targets and drug profiles of screened hits.

| **S. No.** | **Drug** | **Target** | **Profile description** |
| --- | --- | --- | --- |
|  | Oxitropium | Muscarinic acetylcholine receptor M5 | Pulmonary Disease, Chronic Obstructive |
|  | Delamanid | Mycolic acid biosynthesis in mycobacteria | An antibiotic used to treat resistant forms of tuberculosis. |
|  | Fentonium | Muscarinic acetylcholine receptors (mAChRs) | Anticholinergic and antispasmodic atropine derivative |
|  | Radotinib | Tyrosine-protein kinase ABL1 | Radotinib is under investigation for the treatment of Leukemia. |
|  | Bictegravir | Reverse transcriptase/RNaseH;  Integrase | A medication used to treat HIV infections. |
|  | Mosapramine | Dopamine | Atypical antipsychotic drug |
|  | Alectinib | ALK tyrosine kinase receptor | A medication used to treat some forms of lung cancer. |
|  | Fendosal | Cyclooxygenase (COX) enzymes – likely COX-1 and COX-2 | Anti-inflammatory, analgesic |
|  | Pimozide | Dopamine receptor; Calmodulin; Voltage-gated inwardly rectifying potassium channel KCNH2 | A medication used to manage severe tics (when a person makes sudden sounds or movements) in Tourette's Disorder. |
|  | Conivaptan | Vasopressin V1a/V2 receptor | A medication used to increase sodium levels in the blood. |
|  | Selumetinib | Mitogen-activated protein kinase kinase 1 and 2 (MEK1 and MEK2) | Anticancer: A medication used to treat a rare type of cancer in children. |
